# Comparison of VR-Assisted Therapy to Cognitive-Behavioral Therapy in the treatment of verbal hallucinations in patient with treatment-resistant schizophrenia

**DOI:** 10.1192/j.eurpsy.2023.331

**Published:** 2023-07-19

**Authors:** M. Beaudoin, S. Potvin, A. Dumais

**Affiliations:** 1 Psychiatry and addictology, University of Montreal; 2 Research center, Institut universitaire en sante mentale de Montreal; 3 Faculty of Medicine, McGill University; 4 Institut national de psychiatrie légale Philippe-Pinel, Montreal, Canada

## Abstract

**Introduction:**

Auditory verbal hallucinations (AVH), which often present in individuals with schizophrenia, can cause great psychological distress. Although antipsychotic medication can reduce AVH, 30% of patients will be resistant. Treatment resistance could be associated with important consequences, and there are only few treatment alternatives. Unfortunately, more than 50% of patients will not respond favorably to the best available psychological therapy, cognitive-behavioral therapy (CBT). However, using virtual reality (VR), our research team has adapted a therapy whose goal is to recreate the faces and voices of the patients’ persecutory voices as avatars. By interacting with an external representation of their hallucinations, patients implement new strategies to control their voices and to regulate their emotions. A small clinical trial (proof of concept) has demonstrated the safety and validity of this therapy as well as its superiority compared to the usual treatment.

**Objectives:**

(1) To verify whether VR-Assisted Therapy (VRT) is superior to CBT for the treatment of AVH; and (2) to examine the effects of VRT and CBT on the symptomatology and quality of life.

**Methods:**

For this single-blind randomized controlled clinical trial, 136 patients will by recruited (68/intervention) The effects on psychotic symptomatology as well as on quality of life is assessed using standardized questionnaires. Patients are included if they hear persecutory voices and have not responded to at least 2 trials of antipsychotics. Both groups are randomized from an external site and begin with an initial clinical assessment prior to randomization. Participants then receive 9 weekly one-hour therapy sessions (CBT or VRT), beginning the following week. A second clinical evaluation is carried out one week after the last session. A linear mixed effects model will be used to compare the effects of the 2 interventions.

**Results:**

Results from a pilot randomized comparative trial evaluating the short- and long-term efficacy of VRT over CBT for patients with treatment-resistant schizophrenia (N=37/group) showed that both interventions produced significant improvements in AVH severity and depressive symptoms. Although results did not show a statistically significant superiority of VRT over CBT for AVH, VRT did achieve larger effects particularly on overall AVH (*d* = 1.080 for VRT and *d* = 0.555 for CBT). The recruitment of the current clinical trial is in progress.

**Image:**

**Figure:**
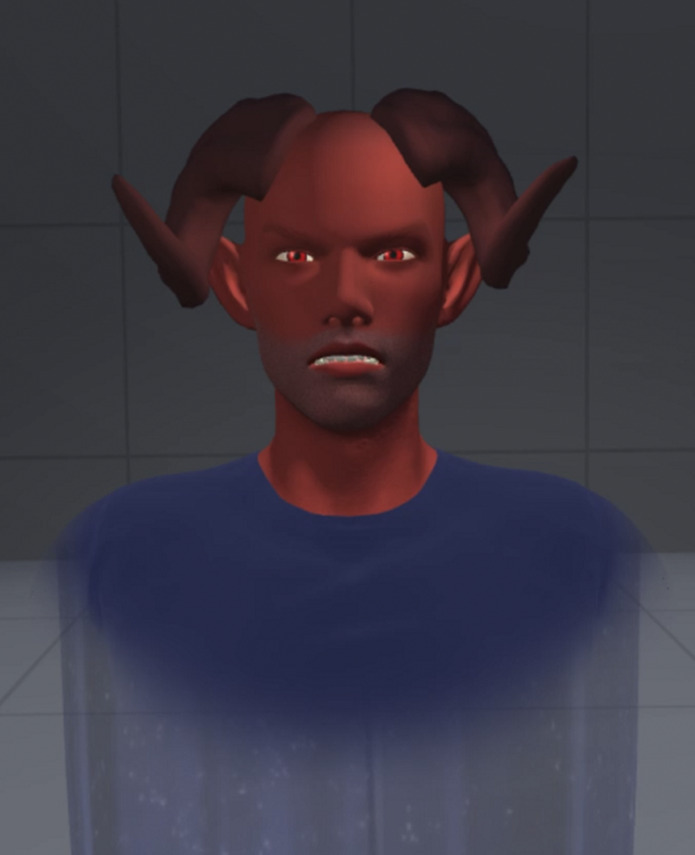

**Conclusions:**

VRT, which is currently under clinical investigation, presents itself as an interesting complement to existing pharmacological treatments. Since VR technologies allow the recreation of scenarios that are nearly impossible to reproduce in real life, VR-assisted psychotherapy could eventually become an integral part of our clinical practices.

**Disclosure of Interest:**

None Declared

